# Simultaneous quantification of venetoclax, selinexor, voriconazole and fluconazole in human plasma by HPLC-MS/MS and its application in hematological malignancies

**DOI:** 10.3389/fphar.2026.1827916

**Published:** 2026-09-11

**Authors:** Feifei Han, Yinying Wang, Yuan Jian, Ting Hu, Ying Zhu, Xian Ding, Jinghui Zhang, Rao Wei, Honghu Zhu, Zhuoling An

**Affiliations:** 1 Department of Pharmacy, Beijing Chao-Yang Hospital, Capital Medical University, Beijing, China; 2 Department of Hematology, Beijing Chao-Yang Hospital, Capital Medical University, Beijing, China; 3 Institute for Cancer, Chinese Institutes for Medical Research (CIMR), Beijing, China; 4 Beijing Key Laboratory of Tumour Resistance Mechanism and Clinical Translation, Beijing, China

**Keywords:** venetoclax, selinexor, voriconazole, fluconazole, HPLC-MS/MS, hematologic malignancies

## Abstract

**Background:**

Venetoclax and selinexor are essential targeted agents for hematological malignancies, but their clinical application is challenged by pronounced ethnic pharmacokinetic differences—especially in Chinese patients—and CYP3A-mediated drug-drug interactions (DDIs) with azole antifungals (voriconazole, fluconazole). No existing LC-MS/MS method simultaneously quantifies all four drugs, creating an unmet need for therapeutic drug monitoring (TDM). We aimed to develop and validate a rapid, sensitive HPLC-MS/MS method for simultaneous quantification of venetoclax, selinexor, voriconazole, and fluconazole in human plasma.

**Methods:**

Plasma samples underwent protein precipitation pretreatment. Chromatographic separation utilized a C18 column with acetonitrile and 0.1% formic acid gradient elution. Mass detection employed positive-ion multiple reaction monitoring (MRM). The method was validated for specificity, linearity, precision, accuracy, recovery, matrix effect, and stability, then applied to AML patients receiving these medications.

**Results:**

Calibration ranges were 40–8000 ng/mL (venetoclax), 5–1000 ng/mL (selinexor), 100–10000 ng/mL (voriconazole), and 320–32000 ng/mL (fluconazole), all with *r* ^2^ > 0.9976. Intra-day and inter-day precision and accuracy met acceptance criteria. No significant matrix effect or carry-over was observed. Selinexor exhibited limited stability (<85% remaining after 4 h at room temperature or 24 h at 4 °C); other analytes were stable throughout. The median venetoclax steady-state trough concentration (Css,trough) in Chinese patients was 1025.08 ng/mL. Co-administration with voriconazole increased venetoclax exposure 1.75–14-fold, whereas fluconazole produced a consistent 2.4–2.9-fold increase. Venetoclax 100 mg/d combined with azole antifungals achieved exposures comparable to or exceeding 400 mg/d monotherapy.

**Conclusion:**

This is the first HPLC-MS/MS method enabling simultaneous quantification of venetoclax, selinexor, voriconazole, and fluconazole, successfully deployed in routine TDM. Voriconazole’s marked DDI variability underscores the necessity of TDM-guided dosing, while fluconazole offers a more predictable antifungal prophylaxis alternative. These results deliver population-specific pharmacokinetic data supporting rational co-administration of venetoclax with azole antifungals in Chinese hematologic malignancy patients.

## 1 Introduction

Hematological malignancies are a heterogeneous group of malignant disorders originating from the hematopoietic and lymphoid systems, with high morbidity and mortality. Clinical management primarily relies on chemotherapy, targeted therapy, and immunotherapy. In recent decades, advances in targeted drug development have revolutionized their treatment, with venetoclax and selinexor emerging as pivotal agents that significantly improve patient prognosis (Kumar et al., 2023; Garfall, 2024; Hu et al., 2023; Eichhorst et al., 2023; Grosicki et al., 2020; Syed, 2019; Kalakonda et al., 2020; Chari et al., 2019).

Venetoclax functions as a highly selective B-cell lymphoma-2 (Bcl-2) inhibitor, inducing tumor cell apoptosis by targeting the anti-apoptotic Bcl-2 protein, and is widely utilized in treating chronic lymphocytic leukemia (CLL), acute myeloid leukemia (AML), and other hematological malignancies. As the first-in-class exportin 1 (XPO1) inhibitor, selinexor suppresses XPO1-mediated nuclear export of tumor suppressor proteins and oncoproteins, thereby inhibiting tumor proliferation and promoting apoptosis, and is indicated for relapsed/refractory multiple myeloma (RRMM) and related disorders. Both agents serve as pivotal therapeutics for hematological malignancies, with accumulating clinical and preclinical evidence supporting their favorable efficacy in t (11; 14)-driven hematologic neoplasms and potential utility in relapsed/refractory cases (Nguyen et al., 2022; Liu et al., 2024; Jiang et al., 2023; Xiao et al., 2024).

Global studies have shown that when venetoclax is combined with hypomethylating agents (HMAs), a dose of 400 mg once daily (QD) can bring the efficacy to a plateau; when combined with Low-Dose Cytarabine, a dose of 600 mg QD achieves the efficacy plateau, and higher doses provide no additional benefit (Kalakonda et al., 2020; Chari et al., 2019). Notably, emerging data indicate significant ethnic differences in venetoclax exposure—critical for Chinese patients, as their exposure differs distinctly from that of other populations reported in existing studies. When healthy Chinese volunteers receive the same dose, their maximum plasma concentration (Cmax) is 94% higher and the area under the concentration-time curve (AUC) is 66% higher than those of non-Asian populations (Garfall, 2024; Eichhorst et al., 2023). This is consistent with observations in Asian AML patients, where venetoclax exposure is twice as high as that in non-Asian patients, potentially altering the drug’s efficacy and safety profiles (Kalakonda et al., 2020). Our previous study on 13 Chinese patients with acute leukemia (10 cases of AML, 3 cases of ALL) showed a median venetoclax plasma concentration of 2030 ng/mL (Yuan et al., 2024).

Venetoclax combined with HMAs often causes prolonged, profound neutropenia, necessitating antifungal prophylaxi s (Rausch et al., 2021; De Gregori et al., 2023). Voriconazole and fluconazole, first-line antifungal agents for immunocompromised hematological patients, can increase plasma concentrations of venetoclax and selinexor, leading to a high risk of drug-drug interactions (DDIs). Additionally, venetoclax, selinexor, voriconazole, and fluconazole exhibit significant interindividual pharmacokinetic variability, influenced by age, gender, liver/kidney function, genetic polymorphism, and concomitant medications. For instance, as CYP3A inhibitors, voriconazole and fluconazole can markedly elevate venetoclax trough concentrations (Gao et al., 2023), increasing the risk of myelosuppression, infection, and other adverse reactions. Given these risks, coupled with the unique venetoclax exposure characteristics of Chinese patients, therapeutic drug monitoring (TDM) of these four drugs is crucial for formulating individualized regimens, ensuring efficacy, and minimizing adverse reactions.

Notably, most existing HPLC-MS/MS methods detect only one or two of the four drugs, with few reports on their simultaneous quantification in human plasma—a critical gap, especially for Chinese hematological malignancy patients receiving combined medication. Establishing a simultaneous detection method can not only simplify the detection process, reduce sample dosage and detection cost, but also provide a more efficient and convenient technical means for TDM in these patients. Therefore, this study aims to establish a simple, rapid, sensitive, and specific HPLC-MS/MS method for the simultaneous determination of venetoclax, selinexor, voriconazole, and fluconazole in human plasma, and to verify its methodological performance, so as to provide reliable technical support for clinical TDM, the formulation of individualized medication regimens, and the improvement of clinical treatment effect in Chinese patients.

## 2 Materials and methods

### 2.1 Reagents and instruments

The reference standard of venetoclax and selinexor (purity: 99%) were provided by MCE; voriconazole and fluconazole (purity>98%) were obtained from TCI, the voriconazole -D3 and fluconazole-D4 was purchased from Cayman Chemical; methanol and acetonitrile (HPLC grade) were obtained from Merck; Milli-Q water purification system was used to provide pure water. Formic acid (mass spectrometry grade) was purchased from Thermo Fisher Scientific (Waltham, United States). Blank human serum/plasma (free of target analytes) was provided by the Clinical Laboratory of Beijing Chaoyang Hospital.

The analytical system consisted of a LC20ADXR HPLC system (SHIMADZU, Japan) and an AB Sciex QTRAP 5500 Qtrap spectrometer (AB Sciex, Framingham, United States). Other instruments included a Mettler Toledo XS205DU analytical balance (Mettler Toledo, Zurich, Switzerland), a Thermo Scientific Sorvall ST16R high-speed refrigerated centrifuge (Thermo Fisher Scientific, Waltham, United States), and a Vortex-Genie two vortex mixer (Scientific Industries, Bohemia, United States).

### 2.2 Chromatographic and mass spectrometric conditions

Chromatographic separation was conducted on a XBridge C18 column (2.1 × 50 mm, 3.5 μm) with a mobile phase consisting of 0.1% formic acid in water (phase A) and 0.1% formic acid in acetonitrile (phase B) under gradient elution: 0–1.0 min, 85% A (hold); 1.0–1.1 min, 85% A→15% A; 1.1–5.1 min, 15% A (hold); 5.1–5.2 min, 15% A→85% A; 5.2–6.0 min, 85% A (stop). Flow rate and injection volume were 0.4 mL/min and 1 μL, respectively.

Electrospray ionization (ESI) in positive ion mode coupled with multiple reaction monitoring (MRM) was utilized for mass spectrometric detection. Key instrumental parameters were optimized and set as follows: the spray voltage was adjusted to 4.5 kV, sheath gas pressure was controlled at 35 psi, auxiliary gas pressure was set to 10 psi, and the ion transfer tube was maintained at 350 °C. Detailed information regarding the optimized MRM transitions and corresponding parameters for each target analyte and its matched internal standard is presented in Table 1.

**TABLE 1:** Selected-reaction monitoring conditions and optimized mass parameters for the immunosuppressant drugs.

Analyte	Precursor ion/product ion (m/z)	Dwell time (ms)	DP(V)	EP(V)	CE(V)	CXP(V)	Function
Venetoclax	868.5/321	100	40	10	50	13	Quantitative ion pair
Venetoclax-d7	875.5/321	100	40	10	50	13	Internal standard
Selinexor	444.1/334	100	110	10	40	13	Quantitative ion pair
Eltanexor	429/409	100	30	10	35	13	Internal standard
Voriconazole	350.1/126.9	100	50	10	5	13	Quantitative ion pair
Voriconazole-d3	353.1/284	100	50	10	10	13	Internal standard
Fluconazole	307.1/238	100	50	10	5	13	Quantitative ion pair
Fluconazole-d4	311.2/242.1	100	107	10	10	13	Internal standard

### 2.3 Sample preparation

Blood samples were delivered to the laboratory within 2 h after collection. If immediate analysis was not feasible, samples were centrifuged to separate plasma, and the obtained plasma was stored at −80 °C until further analysis. Samples with lipemia or hemolysis were excluded from the analysis. A 50 μL aliquot of blood sample (plasma) was placed in a 1.5 mL centrifuge tube, followed by the addition of 10 μL mixed internal standard working solution (100 ng/mL for each internal standard: venetoclax-D7, eltanexor, voriconazole-d3, fluconazole-D4). After vortex mixing for 30 s, 500 μL acetonitrile containing 0.1% formic acid was added, and the mixture was vortexed for 1 min to precipitate proteins. The sample was centrifuged at 13,000 g for 10 min 100 μL of the supernatant was injected into the HPLC-MS/MS system for analysis.

### 2.4 Method validation

Bioanalytical method validation was implemented in strict compliance with the Guidance for Industry Bioanalytical Method Validation issued by the FDA (2018) and the Guideline on Bioanalytical Method Validation released by the NMPA (2020) (Bioanalytical Method Validation, 2018; NMPA Guideline on Bioanalytical Method Validation, 2020). The validation process focused on several critical performance parameters, which are described in detail below:

#### 2.4.1 Accuracy and precision

Quality control (QC) samples were prepared at the predefined concentrations for each analyte, namely 40 ng/mL for venetoclax, 5 ng/mL for selinexor, 100 ng/mL for voriconazole, and 160 ng/mL for fluconazole. Intra-day precision and accuracy were assessed by analyzing six replicate samples per day over three consecutive days, whereas inter-day precision and accuracy were determined based on a total of 18 replicates (6 replicates per day × 3 days). The validation was considered acceptable if the accuracy ranged between 85% and 115% (with a range of 80%–120% for the lower limit of quantification [LLOQ]) and the coefficient of variation (CV) was ≤15% (≤20% for LLOQ).

#### 2.4.2 Recovery

QC samples and blank matrix spiked with analytes after extraction were detected. Recovery = (peak area of QC sample/peak area of post-extraction spiked sample) × 100%. Six replicates were tested for each analyte. Acceptance criteria: recovery 85%–115% with CV ≤ 15%.

#### 2.4.3 Matrix effect

IS-normalized matrix factor was calculated as (peak area ratio of analyte to IS in spiked blank matrix/peak area ratio of analyte to IS in standard solution) × 100%. Six replicates of blank matrix from different donors were used. Acceptance criteria: matrix factor 85%–115% with CV ≤ 15%.

#### 2.4.4 Stability

The stability of analytes in blood samples was evaluated under five common storage and handling conditions: room temperature for 4 h (simulating sample standing before processing), 4 °C for 4 h and 24 h (simulating short-term storage in a refrigerator), −80 °C for 30 days (simulating long-term storage), and three freeze-thaw cycles (simulating repeated sample thawing). QC samples were prepared and detected under each condition, with accuracy and precision used as evaluation indicators. Acceptance criteria: accuracy 85%–115%, CV ≤ 15%.

#### 2.4.5 Data analysis

All data were analyzed using Analyst 1.6.3 software (AB Sciex) and GraphPad Prism 9.0 software (GraphPad Software, San Diego, USA). Quantitative analysis was performed using the internal standard method with weighted least squares linear regression (weight = one/x^2^).

### 2.5 Patients and drug administration

Adult men and women diagnosed with histologically confirmed AML by World Health Organization criteria who are undergoing venetoclax treatment are included in this study. Venetoclax was administrated with a constant dose for at least 3 days. Blood samples for determination of venetoclax and voriconazole/Fluconazole concentrations were collected using potassium EDTAcontaining tubes by venipuncture before 0.5 h (Cmin) of venetoclax dosing (Rausch et al., 2021; De Gregori et al., 2023). The ethical principles of this study were conducted in accordance with the Declaration of Helsinki and Good Clinical Practice guidelines. This study was approved by the Ethics Committee of Beijing Chaoyang Hospital.

### 2.6 Statistical analysis

Statistical analysis was performed using GraphPad Prism software, and Mann-Whitney non-parametric test was applied for inter-group comparison of blood drug concentrations.

## 3 Results

### 3.1 Selectivity and carry-over

**FIGURE 1:**
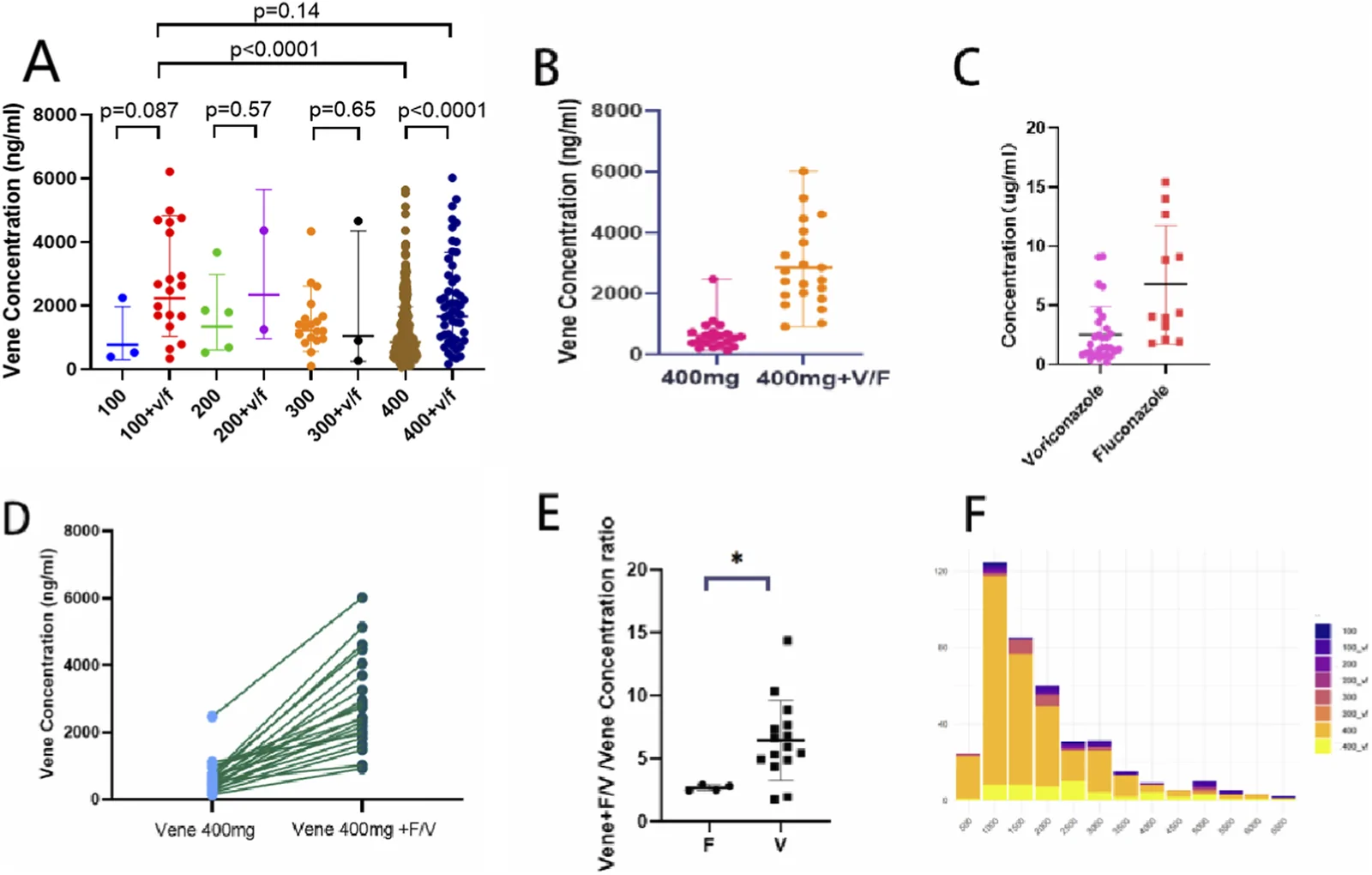
Distribution of venetoclax trough concentrations of venetoclax concentrations **(A)** Distribution of the trough concentrations of venetoclax when administered alone at different doses versus in combination with voriconazole or fluconazole, **(B)** Distribution of venetoclax trough concentrations in the same patients receiving 400 mg venetoclax alone versus 400 mg venetoclax in combination with voriconazole or fluconazole, **(C)** Concentration distribution of voriconazole and fluconazole when co-administered with venetoclax, **(D)** Comparison of venetoclax trough concentrations in the same patients within the same treatment cycle before and after the addition of voriconazole or fluconazole to venetocthe distribution of venetoclax concentrations under different dosing regimenslax therapy, **(E)** The concentration ratio of venetoclax (steady-state) in combination with voriconazole or fluconazole versus monotherapy, **(F)** The distribution of venetoclax concentrations under different dosing regimens.

**FIGURE 2:**
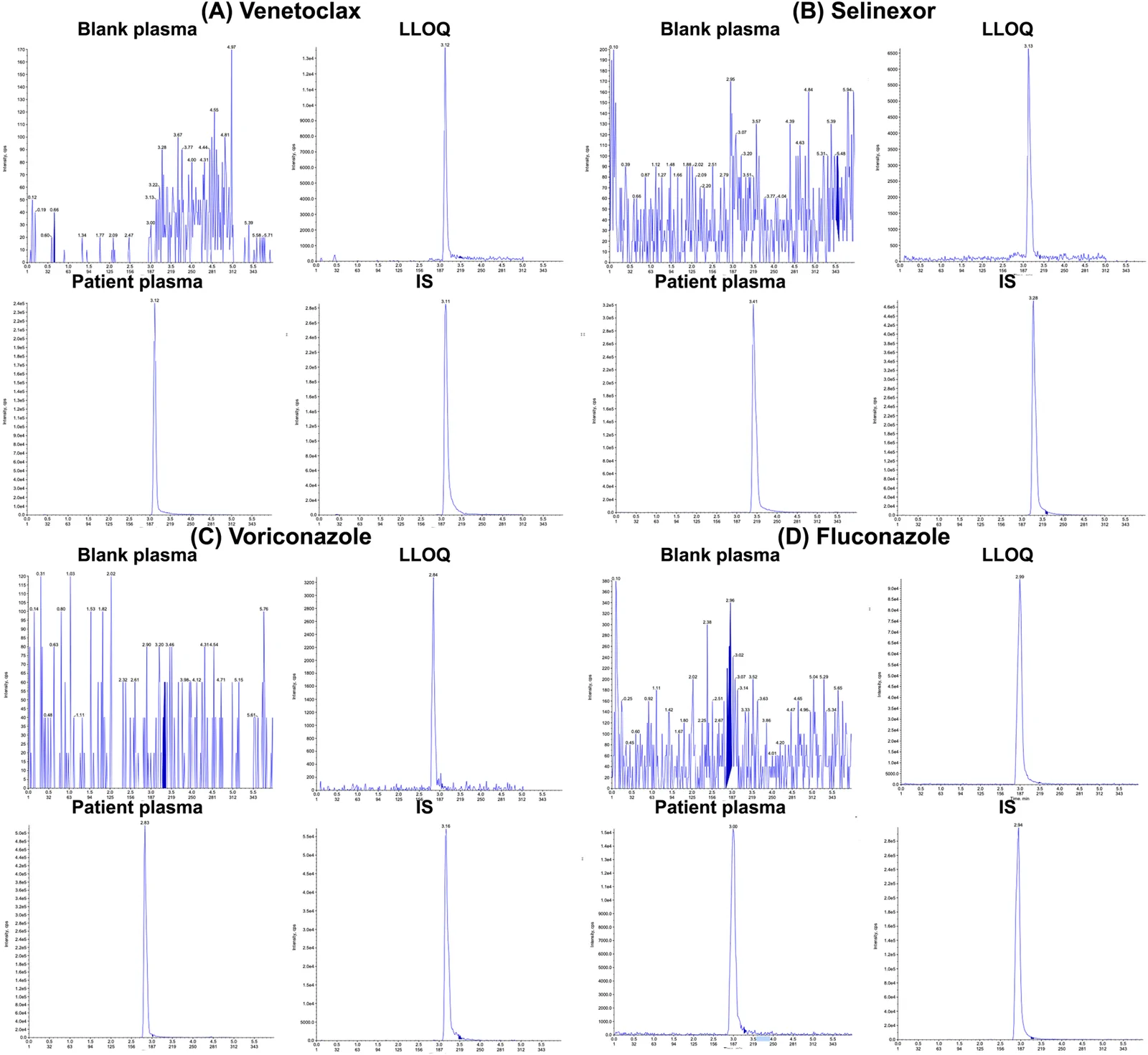
Representative chromatograms of blank plasma sample, LLOQ, patient plasma and IS: **(A)** venetoclax, **(B)** selinexor, **(C)** voriconazole, **(D)** fluconazole.

Six batches of human blank plasma from six different sources were analyzed to verify the selectivity. The response of interfering components less than 20% of the lower limit of quantification (LLOQ) for the analyte and 5% for IS was accepted. The typical chromatograms were shown in Figure 1. The retention times of venetoclax, venetoclax-d7, selinexor, eltanexor, voriconazole, voriconazole-D3, fluconazole and fluconazole-d4 were approximately 3.6–3.8 min, 3.5–3.7 min, 3.4–3.6 min and 3–3.3 min respectively (Figure 2). No remarkable endogenous interference was observed around the retention time. Blank samples were analyzed after successive injection of the upper limit of quantification (ULOQ) sample. The response of analytes in blank sample was compared with that of LLOQ to evaluate the carry-over. The response of carry-over was in an acceptable range.

### 3.2 Linearity and LLOQs

The linearity was determined by analyzing at least six non-zero concentrations of venetoclax, selinexor, and six non-zero concentrations voriconazole, fluconazole and then the linear regression equation was calculated by the weighted least squares method with one/x^2^ as the weighing factor. The calibration range was 40–8000 ng/mL for venetoclax and 5–1000 ng/mL for Selinexor, 100–10000 ng/mL for voriconazole and 320–32000 ng/mL for fluconazole, which is capable for measuring the concentration of these four analytes according to the published pharmacokinetic study. Three replicate regression curve was evaluated, with correlation coefficients (r^2^)>0.9976 at least, including the LLOQ, which indicated a good fit of the regression equation.

### 3.3 Precision and accuracy

Six replicate LLOQ or QC samples were analyzed in three sequential days to investigate intra-day and inter-day accuracy and precision. The relative standard deviation RSD (%) was used to evaluate the precision, which should not be more than 15%. The relative error (RE) was used to measure the accuracy, which was expected within ±15% for QC levels, and ±20% for LLOQ samples. The intra-day and inter-day precision of venetoclax, selinexor, voriconazole and fluconazole all concentrations were shown in Table 2. All the precision and accuracy can meet the acceptance criteria of FDA guidelines.

**TABLE 2:** Accuracy, intra-day and inter-day precision, extraction recovery, and matrix factor results for Venetoclax, Selinexor, voriconazole and Fluconazole.

Analyte	Concentration (ng/mL)	Intraday 1 (n = 6)		Intraday 2 (n = 6)		Intraday 3 (n = 6)		Inter-day (n = 18)		Recovery (n = 6)		IS-normalized matrix factor (n = 6)	
		Accuracy (%)	Precision (CV%)	Accuracy (%)	Precision (CV%)	Accuracy (%)	Precision (CV%)	Accuracy (%)	Precision (CV%)	Accuracy (%)	Precision (CV%)	Accuracy (%)	Precision (CV%)
Venetoclax	40	91.03	4.65	97.00	4.84	96.23	2.35	94.76	4.80	-	-	-	-
	120	90.05	4.07	91.45	4.03	89.85	4.03	90.45	3.45	88.50	2.17	87.36	1.67
	4000	93.55	3.46	92.73	2.43	90.82	4.33	92.37	3.51	93.00	1.86	92.23	3.90
	6000	101.72	1.63	98.63	2.30	99.77	2.11	100.04	2.31	91.71	3.27	91.53	3.25
Selinexor	5	93.28	4.28	95.48	2.92	99.93	1.24	96.23	4.09	-	-	-	-
	15	101.27	3.86	96.70	2.68	106.83	3.04	101.60	5.19	88.73	1.72	99.33	2.43
	500	87.12	1.26	90.40	1.44	89.60	1.79	89.04	2.22	96.67	2.56	108.00	2.33
	750	91.23	1.52	100.95	1.29	100.12	1.80	99.15	2.51	99.60	1.76	104.66	1.40
Voriconazole	100	107.83	7.98	100.42	4.48	106.67	4.95	104.97	6.54	-	-	-	-
	200	94.28	4.57	99.50	1.67	96.03	3.37	96.61	3.92	91.33	4.42	88.51	5.44
	5000	100.22	1.24	103.50	1.59	100.25	1.26	101.32	2.03	95.87	1.01	93.85	2.56
	7500	101.35	1.58	103.17	1.13	98.65	2.40	101.06	2.51	95.13	1.41	96.74	2.60
Fluconazole	320	102.62	4.75	111.00	2.35	96.63	6.43	103.42	7.31	-	-	-	-
	640	97.35	3.94	108.67	4.10	95.35	4.08	100.46	7.11	93.25	7.72	91.96	3.72
	16,000	97.00	2.83	109.83	3.02	99.30	2.58	102.04	6.23	102.65	8.96	99.50	3.48
	24,000	97.70	2.44	108.83	2.35	99.42	1.55	101.98	5.34	107.40	9.91	100.19	4.94

### 3.4 Matrix effect and recovery

Six blank plasma samples collected from six individual donors were prepared by the method mentioned above, then standard working solutions of venetoclax, selinexor, voriconazole and fluconazole were added, and the ratio of the corresponding peak area between that of the standards at equivalent concentration was defined as the matrix effect (ME). The normalization factor, which calculated as the ratio of the matrix factor of analytes and IS was used to evaluate the matrix effects. The extraction recovery of analytes was calculated by dividing the extracted QC samples mean response with that from standard solutions added into post-extracted blank human plasma at the same concentrations.

The results of extraction recovery and matrix effect for these analytes are shown in Table 2.

### 3.5 Stability

The QC samples were used to investigate the stability of venetoclax, selinexor, voriconazole and fluconazole in plasma at the following conditions: storage at − 80 °C for 30 days, after three freeze/thaw cycles, 4 h at room temperature, 4 °C for 4 h and 24 h. As shown in Table 3, no obvious degradation was observed in venetoclax, voriconazole and fluconazole under the variety of situations. The stability of selinexor is unsatisfactory; less than 85% of the drug remains following 4 h at room temperature or 24 h at 4 °C. The evaluated conditions can cover the status of clinical samples throughout the testing process, ensuring the results are authentic and credible. The poor stability of selinexor imposes certain limitations on the application of its blood concentration monitoring—blood samples require refrigeration, and long-term storage at −80 °C is necessary when immediate testing is not feasible.

**TABLE 3:** Stability of Venetoclax, Selinexor, voriconazole and Fluconazole under different storage conditions.

Analyte	Spiked (ng mL−1)	Condition	Accuracy (%)	Precision (CV%)
Venetoclax	Low	Room temperature for 4 h	88.46	2.29
		4 °C for 4 h	98.40	7.68
		4 °C for 24 h	91.94	6.91
		−80 °C for 30 days	99.41	4.25
		Three freeze–thaw cycles	104.94	3.96
	Middle	Room temperature for 4 h	104.33	3.74
		4 °C for 4 h	97.22	2.71
		4 °C for 24 h	107.39	3.13
		−80 °C for 30 days	104.93	2.85
		Three freeze–thaw cycles	94.59	2.91
	High	Room temperature for 4 h	102.58	2.60
		4 °C for 4 h	111.91	1.63
		4 °C for 24 h	106.20	2.23
		−80 °C for 30 days	97.07	2.16
		Three freeze–thaw cycles	96.17	1.69
Selinexor	Low	Room temperature for 4 h	68.67	3.02
		4 °C for 4 h	102.43	3.19
		4 °C for 24 h	87.44	1.39
		−80 °C for 30 days	93.35	5.33
		Three freeze–thaw cycles	97.01	2.58
	Middle	Room temperature for 4 h	64.66	1.73
		4 °C for 4 h	104.25	1.03
		4 °C for 24 h	78.58	2.53
		−80 °C for 30 days	97.92	0.97
		Three freeze–thaw cycles	96.11	1.45
	High	Room temperature for 4 h	66.37	2.37
		4 °C for 4 h	108.98	1.75
		4 °C for 24 h	77.06	0.55
		−80 °C for 30 days	99.12	1.73
		Three freeze–thaw cycles	101.41	1.56
Voriconazole	Low	Room temperature for 4 h	106.55	3.77
		4 °C for 4 h	103.04	5.38
		4 °C for 24 h	108.58	3.90
		−80 °C for 30 days	105.94	4.11
		Three freeze–thaw cycles	96.86	5.76
​	Middle	Room temperature for 4 h	105.45	3.40
		4 °C for 4 h	108.02	1.92
		4 °C for 24 h	108.95	2.25
		−80 °C for 30 days	94.88	4.00
		Three freeze–thaw cycles	92.59	1.64
	High	Room temperature for 4 h	101.94	4.15
		4 °C for 4 h	108.73	4.04
		4 °C for 24 h	102.81	3.41
		−80 °C for 30 days	94.06	3.34
		Three freeze–thaw cycles	90.92	3.23
Fluconazole	Low	Room temperature for 4 h	102.28	4.45
		4 °C for 4 h	94.12	6.86
		4 °C for 24 h	93.60	5.99
		−80 °C for 30 days	94.20	9.51
		Three freeze–thaw cycles	106.15	4.81
	Middle	Room temperature for 4 h	106.09	7.13
		4 °C for 4 h	103.64	5.71
		4 °C for 24 h	96.48	4.32
		−80 °C for 30 days	95.64	4.74
		Three freeze–thaw cycles	103.68	3.44
	High	Room temperature for 4 h	96.84	5.46
		4 °C for 4 h	94.58	6.00
		4 °C for 24 h	100.04	5.54
		−80 °C for 30 days	103.26	1.93
		Three freeze–thaw cycles	98.24	1.84

### 3.6 Method application

After validation, the HPLC-MS/MS approach was used in venetoclax, voriconazole and fluconazole measure in 386 blood drug concentration monitoring sessions at Beijing Chao-Yang Hospital (Table 4). EDTA blood amples were collected and sent to the laboratory within 2 h. Whole blood samples were then preprocessed and analyzed according to the method described above. The results were exhibited in Figure 1. We could get some information as follows:

**TABLE 4:** Patients and treatment characteristics.

Characteristics	VENE 100 mg + V/F	VENE 400 mg	VENE 400 mg + V/F
Sex	Female 5; Male 13	Female 142; Male 142	Female 17; Male 35
Age (year)	58 (47.5–67.75)	64 (50–69)	60 (50–69.75)
Weight (kg)	70 (60–74)	61 (67–74.6)	67 (62–75.4)
BMI (kg/m2)	24.03 (21.6–25.01)	24.91 (22.08–26.4)	25.15 (23.44–26.66)
Azoles	200 mg q12 h V/0.15 g qd F	-	200 mg q12 h V/0.15 g qd F
Steady-state trough concentration	2630 (1670–4622)	861.6 (493.3–1532)	1886 (1004–2924)

This study investigated the plasma concentrations of venetoclax following multiple administrations at different doses, both alone and in combination with voriconazole or fluconazole. Results are presented as median and interquartile range (IQR). All data were derived from original statistical analyses, with detailed group-specific results as follows: Group 100 (venetoclax 100 mg alone, three patients): Median (IQR) = 524 (391.2–2240); Group 100 + v/f (venetoclax 100 mg + voriconazole/fluconazole, 19 patients): Median (IQR) = 2630 (1670–4622); Group 200 (venetoclax 200 mg alone, five patients): Median (IQR) = 1789 (600.9–2760); Group 200 + v/f (venetoclax 200 mg + voriconazole/fluconazole, two patients): Median (IQR) = 2806 (1250–4363); Group 300 (venetoclax 300 mg alone, 19 patients): Median (IQR) = 1339 (957.8–1663); Group 300 + v/f (venetoclax 300 mg + voriconazole/fluconazole, three patients): Median (IQR) = 899 (272–4660); Group 400 (venetoclax 400 mg alone, 302 patients): Median (IQR) = 861.6 (493.3–1532); Group 400 + v/f (venetoclax 400 mg + voriconazole/fluconazole, 53 patients): Median (IQR) = 1886 (1004–2924). In conclusion, the median steady-state trough concentration (Css, trough) of venetoclax in the Chinese population is 1025.08 (574.17–2004.5) ng/mL. As CYP3A inhibitors, voriconazole and fluconazole significantly increased the trough concentration of venetoclax: the trough concentration of 100 mg/d venetoclax combined with voriconazole/fluconazole was comparable to that of 400 mg/d venetoclax alone (Figure 1A), with the former [2630 (1670–4622)] being significantly higher than the latter [861.6 (493.3–1532)] and showing no significant difference from 400 mg/d venetoclax combined with voriconazole/fluconazole [1886 (1004–2924)].

No significant differences were observed in the 100 mg, 200 mg, 200 + v/f, 300 mg, and 300 + v/f dose groups, which might be due to the small sample size of these groups, making it insufficient to draw valid conclusions. In general, there were significant differences in blood concentration distribution among different dose groups, and the IQR of venetoclax combined with voriconazole/fluconazole was generally higher than that of the corresponding single-drug groups.

For the same patients, co-administration of venetoclax with voriconazole or fluconazole significantly increased the trough concentration of venetoclax compared with 400 mg/d venetoclax alone (Figures 1B,C). Furthermore, voriconazole and fluconazole exhibited substantial inter-individual variability in their concentrations when co-administered with venetoclax, indicating the necessity of concurrent therapeutic drug monitoring (TDM) for these two agents (Figure 1D). At the same time, these data analysis revealed that voriconazole and fluconazole exerted distinct effects on the plasma concentrations of venetoclax. Analysis of these data revealed that, in the same patients, co-administration with voriconazole increased venetoclax plasma concentrations by 1.75–14-fold, whereas the effect of fluconazole was relatively stable, ranging from 2.4 to 2.9-fold. These findings indicate that voriconazole exerts a more pronounced and variable effect on venetoclax plasma levels, while the impact of fluconazole is more moderate and consistent (Figure 1E).

## 4 Discussion

In this study, we have successfully developed and fully validated a novel, rapid, and sensitive HPLC-MS/MS method for the simultaneous quantification of venetoclax, selinexor, voriconazole, and fluconazole in human plasma. Compared to the widely used immunoassays for therapeutic drug monitoring (TDM), liquid chromatography-tandem mass spectrometry (LC-MS/MS) offers superior flexibility. Through rigorous method validation, LC-MS/MS can be adapted to monitor a broad spectrum of analytes. For drugs with limited patient populations, such as venetoclax and selinexor, for which commercial immunoassay kits are not available, LC-MS/MS enables the development of tailored analytical panels. This capability facilitates clinical TDM and the investigation of drug-drug interactions. To the best of our knowledge, this is the first HPLC-MS/MS approach described that enables the parallel determination of these four compounds in a single run. Compared with previously published methods, our established protocol offers superior speed, sensitivity, and cost-efficiency, making it highly suitable for routine therapeutic drug monitoring (TDM) in clinical settings, particularly for patients with acute myeloid leukemia (AML) and other hematologic malignancies who frequently receive combined regimens of venetoclax, selinexor, voriconazole, and fluconazole.

Selinexor exhibits poor stability, with a room-temperature stability of only 2 hours. This characteristic significantly limits its clinical application, necessitating strict control of the time at room temperature. After sample collection, plasma must be separated promptly upon arrival at the laboratory and either stored at −80 °C or analyzed immediately.

Notably, our pharmacokinetic analysis revealed that co-administration with voriconazole markedly increased venetoclax exposure. Specifically, the peak concentration of venetoclax at 100 mg once daily was elevated to a level comparable to that of 400 mg monotherapy, while its steady-state trough concentration was significantly higher than that observed with 400 mg daily monotherapy. These findings highlight the substantial CYP3A-mediated drug–drug interaction (DDI) potential of voriconazole and emphasize the need for careful dose adjustment in clinical practice.

Consistent with previous research, our results also confirm that azole antifungals significantly enhance venetoclax plasma concentrations (Kumar et al., 2023; Garfall, 2024). Moreover, we observed a striking difference between voriconazole and fluconazole: voriconazole increased venetoclax exposure by 1.75–14-fold, whereas fluconazole produced a more consistent and moderate elevation (2.4–2.9-fold). This variability is likely attributable to the distinct pharmacokinetic properties of the two inhibitors: voriconazole is a potent, time-dependent CYP3A inhibitor with nonlinear kinetics and wide interindividual variability, whereas fluconazole acts as a moderate, more predictable CYP3A inhibitor (Hu et al., 2023; Eichhorst et al., 2023). Clinically, this distinction suggests that fluconazole may facilitate standardized venetoclax dosing, while voriconazole requires individualized TDM to balance efficacy and toxicity.

Furthermore, the median steady-state trough concentration of venetoclax in our Chinese population (1025.08 ng/mL) is consistent with previously reported ranges in Asian cohorts but differs from Western populations, potentially reflecting ethnic differences in CYP3A activity and body composition (Grosicki et al., 2020). Importantly, our data demonstrate that venetoclax 100 mg/d combined with voriconazole/fluconazole achieves exposures similar to or even higher than those of 400 mg/d monotherapy. This observation provides quantitative evidence supporting the need for substantial venetoclax dose reductions when combined with strong/moderate CYP3A inhibitors in Chinese patients.

No significant difference in blood drug concentration was found between the 100 mg + f/v and 400 mg + f/v groups, and the 400 mg + f/v group yielded a lower median concentration. This counterintuitive dose-concentration discrepancy may result from interindividual metabolic heterogeneity and physician individualized dosing decisions. Differences in genetics, hepatorenal function and comorbid conditions may considerably affect drug metabolism. Patients with relatively slow metabolism may achieve higher blood drug exposure at low doses, whereas those with faster drug clearance may exhibit lower drug concentrations despite high-dose treatment. Furthermore, the non-randomized dosing design may partly account for this phenomenon. Clinicians may prescribe 100 mg + f/v to patients with presumed slow metabolism or elevated baseline drug levels to avoid excessive drug accumulation, while 400 mg + f/v may be administered to patients anticipated to have rapid metabolism to ensure adequate therapeutic concentration. Taken together, interindividual metabolic variability and personalized clinical dosing may explain the comparable drug levels between the two groups and the lower median concentration in the 400 mg + f/v group. These findings suggest that blood drug concentration cannot be simply determined by administration dosage, supporting the essential role of therapeutic drug monitoring for individualized dose optimization.

Several limitations of this study should be acknowledged. First, the small sample size in certain dose groups may have contributed to the absence of significant differences, thereby limiting the generalizability of our findings. Second, the combined analysis of voriconazole and fluconazole groups, driven by limited individual patient data, may have obscured subtle differences in their DDI profiles. Third, we did not evaluate the relationship between venetoclax exposure and clinical outcomes, which would further strengthen the clinical utility of our TDM recommendations. Future studies with larger cohorts, separate analysis of each azole, and exposure–response modeling are warranted to address these limitations.

In conclusion, our study provides the first comprehensive characterization of venetoclax trough concentration in Chinese patients and quantifies the differential DDI effects of voriconazole and fluconazole. The marked variability in voriconazole-mediated DDIs underscores the necessity of TDM for personalized venetoclax dosing. When antifungal prophylaxis is clinically indicated, fluconazole may offer a more predictable alternative. Collectively, these findings fill an important gap in population-specific pharmacokinetic data and support the rational, safe, and effective use of venetoclax in combination with azole antifungals among patients with hematologic malignancies.

## Data Availability

The data analyzed in this study is subject to the following licenses/restrictions: The data that support the findings of this study are not publicly available due to privacy and ethical restrictions. The dataset contains confidential clinical information and sensitive patient data that cannot be shared publicly to protect participant privacy. Data are available from the corresponding authors upon reasonable request and with approval of the ethics committee. Requests to access these datasets should be directed to hanfeifei@163.com.
